# Case Report: Tracheobronchomalacia after a two-stage surgical approach to treat congenital diaphragmatic hernia with hepatopulmonary fusion

**DOI:** 10.3389/fped.2025.1573827

**Published:** 2025-07-28

**Authors:** Juri Kanda, Takeo Mukai, Chiharu Irisa, Kyosuke Ibi, Rina Matsuda, Kaori Morita, Akio Ishiguro, Masako Ikemura, Yosuke Yamada, Hisaya Hasegawa, Jun Fujishiro, Naoto Takahashi

**Affiliations:** ^1^ Department of Pediatrics, The University of Tokyo Hospital, Tokyo, Japan; ^2^ Department of Pediatric Surgery, The University of Tokyo Hospital, Tokyo, Japan; ^3^ Department of Pathology, The University of Tokyo Hospital, Tokyo, Japan; ^4^ Department of Neonatology, Tokyo Women's Medical University Adachi Medical Center, Tokyo, Japan

**Keywords:** case report, congenital diaphragmatic hernia, hepatopulmonary fusion, tracheobronchomalacia, neonate

## Abstract

Hepatopulmonary fusion (HPF) is a rare comorbidity of right-sided congenital diaphragmatic hernia (CDH). The mortality rate of CDH with HPF is high, and the optimal approach and timing for surgical intervention remain unclear. Previous studies have reported the importance of avoiding massive intraoperative bleeding, managing pulmonary hypertension, and evaluating abnormal vascular communication in the management of CDH with HPF. However, airway involvement has rarely been documented. Here, we report a case of tracheobronchomalacia (TBM) following a two-stage surgical approach to treat CDH with HPF. The patient was diagnosed with right-sided CDH during gestation. CDH repair was attempted on day of life (DOL) 2. HPF was found intraoperatively, but was left unseparated to avoid massive bleeding. After excluding abnormal vascular communication on computed tomography, a second surgery was performed on DOL 28. HPF was successfully separated, and the right thoracic and abdominal cavities were separated using a polytetrafluoroethylene patch. The patient was extubated on DOL 41, but from DOL 80, the patient started to have cyanotic spells after crying. Because noninvasive positive airway pressure was not effective in preventing these cyanotic spells, a tracheostomy was performed on DOL 133. The results of postoperative bronchoscopy were consistent with TBM. The patient was discharged on home mechanical ventilation and was maintained in a stable respiratory condition. Evaluating pre-and postsurgical anatomical and physiological characteristics is critical in managing CDH with HPF. Our case highlights the importance of assessing airway involvement in such patients.

## 1 Introduction

Hepatopulmonary fusion (HPF) is a rare anomaly occurring in approximately 0.3% of newborns with right-sided congenital diaphragmatic hernia (CDH) (1). As of 2024, up to 40 cases have been reported (2).

HPF is typically diagnosed intraoperatively. The reported mortality rate is 40%–50%. No consensus on the best surgical approach has been established to date (2). Some studies have reported favorable outcomes without complete separation of the lungs and liver, to avoid surgical complications; however, whether complete or partial separation leads to better outcomes remains controversial (1). Furthermore, the optimal timing of surgery remains unclear. A two-stage surgical approach has been reported to be successful in three cases, but the clinical course is highly variable (3–5).

Massive intraoperative bleeding, pulmonary hypertension, and cardiovascular abnormalities have attracted attention in the management of CDH with HPF (1, 2). However, airway involvement has rarely been documented. Here, we report a case of tracheobronchomalacia (TBM) that developed after using a two-stage surgical approach to treat CDH with HPF.

## 2 Presentation of case

The patient was a male infant. Prenatal ultrasonography and magnetic resonance imaging revealed that he had a right-sided CDH, with a lung-to-head ratio of 0.5–0.6.

The patient was born via an elective cesarean section at 38 weeks and 5 days of gestation, with a birth weight of 2,592 g. He was immediately intubated, before the first cry, and was manually ventilated with 100% oxygen and nitric oxide. A narcotic analgesic and a muscle relaxant were administered intravenously. Ten minutes after birth, pre- and postductal oxygen saturation levels were above 90%. His Apgar scores were five and six at 1 and 5 min, respectively.

**Figure 1:**
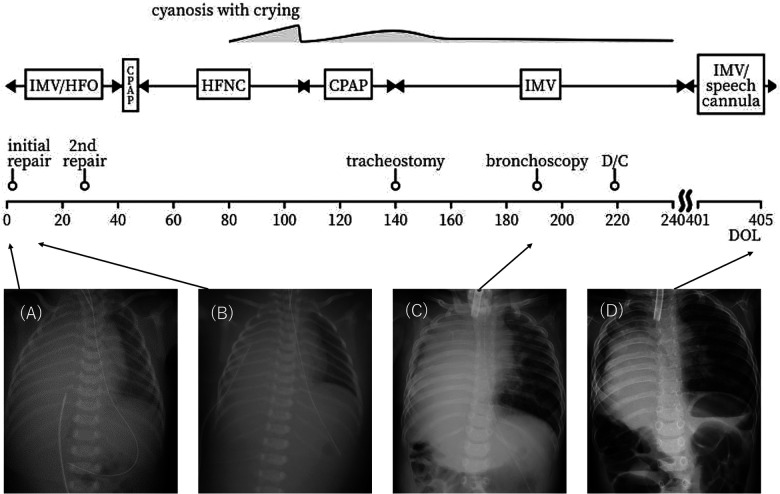
Clinical timeline and chest x-rays. **(A)** Day of life (DOL) 0. The right thorax is almost completely opacified. No evidence of a mediastinal shift is observed. **(B)** DOL 3 (a day after the first operation). The liver remains elevated. **(C)** Six months old. The right lung remains hypoplastic. There is no visualization of intestinal herniation to the thorax. **(D)** Six months after discharge. The right lung volume has increased. Managed with home mechanical ventilation, but gradually spends more time with a speech valve. IMV = intermittent mandatory ventilation HFO = high frequency oscillations CPAP = continuous positive airway pressure HFNC = high flow nasal cannula D/C = discharge.

Chest radiography performed on admission revealed near-complete opacification of the right hemithorax (Figure 1A). Echocardiography showed a patent ductus arteriosus, a patent foramen ovale, or a small atrial septal defect with bidirectional shunting, and 1.8 m/s tricuspid regurgitation.

**Figure 2:**
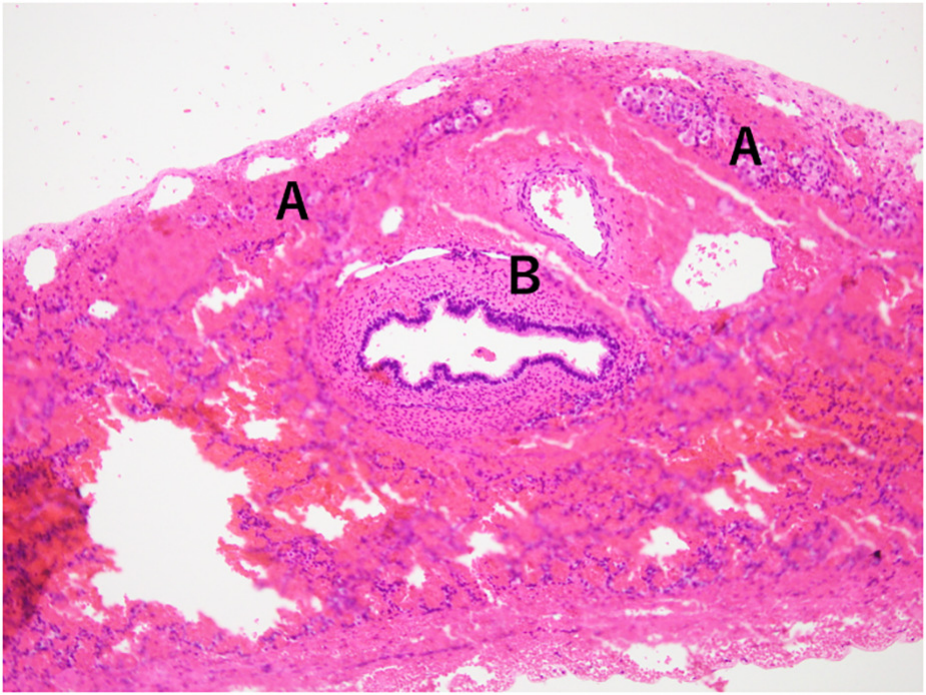
Histopathology of hernia sac specimen showing hepatocytes **(A)** and bronchi **(B)**, without a plane of separation.

The initial CDH repair was attempted on day of life (DOL) 2 using an abdominal approach through a right subcostal incision. The right lobe of the liver, the gallbladder, and part of the bowel were found to be herniated into the right thoracic cavity. The herniated bowel could be reduced to the abdominal cavity, whereas the liver was immovable. Therefore, an additional thoracotomy through the ninth intercostal space was performed. After careful inspection from both the thoracic and abdominal sides, the extremely hypoplastic right lung and the liver were found to be tightly fused via a hernial sac without a safe surgical plane. Since this inspection led to pulmonary hemorrhage, the lung and the liver were left unseparated to avoid severe hemorrhage. The CDH was classified as type C according to the Congenital Diaphragmatic Hernia Study Group classification (6). Part of the hernial sac that was not adhered to the lung or liver was resected for pathological examination (Figure 2). The results were consistent with those of HPF. The outer hernial sac was sutured as much as possible to prevent intestinal prolapse. Subsequent x-ray findings showed no obvious intestinal protrusion into the thoracic cavity (Figures 1B–D).

Postoperatively, nitric oxide was successfully withdrawn; however, the patient could not be weaned from mechanical ventilation. Postoperative computed tomography (CT) showed no aberrant vessel connections between the lungs and liver. The second surgical session to repair the CDH with the HPF was performed on DOL 28. The second surgical session to repair the CDH with HPF was performed on DOL 28 using a right thoracic approach through the fifth subcostal incision. During this session, the surgeons carefully separated the fused right lung and liver with minimal resection of the trachea and lung. Superficial bleeding of the liver and lung air leakage were unavoidable but were controlled with TachoComb®. After reducing the liver to the abdomen, the right thoracic and abdominal cavities were separated with a GORE-TEX® soft tissue patch.

The patient was extubated on DOL 41 and weaned from nasal continuous positive airway pressure (CPAP) to high-flow nasal cannula on DOL 48. Oral feeding was initiated on DOL 63. From approximately DOL 80 onwards, the patient experienced high-pitched wheezes and cyanotic spells with bradycardia after crying. Gradually, the patient developed a feeding aversion.

Perfusion lung scintigraphy on DOL 80 revealed that the right lung contributed only 3.3% of the total lung perfusion. According to postoperative echocardiography, pulmonary artery pressure was not high.

The patient was administered nasal CPAP again from DOL 106 onward. Wheezing and cyanotic spells resolved after escalation to nasal CPAP; however, his symptoms gradually worsened again.

**Figure 3:**
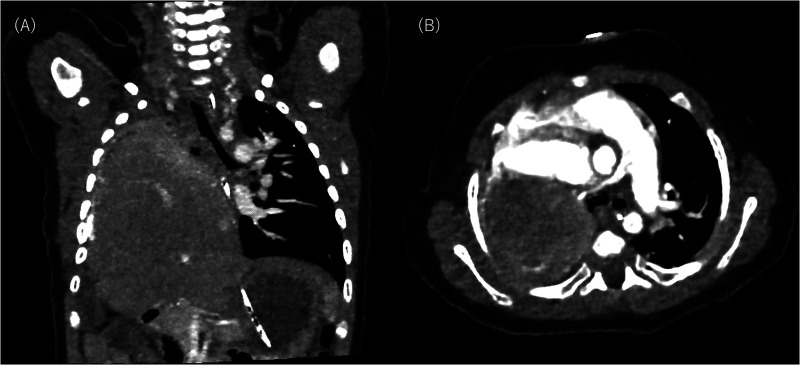
Postoperative computed tomography scan on day of life 133: **(A)** shows coronal slice and **(B)** shows axial slice. The right lung and pulmonary vessels are hypoplastic. No aberrant vessel communication is seen between the right lung and the liver. The liver remains elevated in the right thorax without compressing the trachea and the left bronchus.

As postoperative CT conducted on DOL 133 did not indicate tracheal or left bronchial compression by the liver (Figure 3), the surgeons considered that it would not be feasible to surgically move the liver downward. Instead, as the clinical course suggested severe tracheomalacia (TM)/TBM, a tracheostomy was proposed to secure a safe airway, prevent airway collapse by generating stable high positive end-expiratory pressure, and prevent excessive work of breathing with mechanical ventilation. The parents initially refused and wanted their child to remain hospitalized until he no longer needed respiratory support. They expressed concerns about their child losing his voice, about raising a child with a tracheostomy at home, and about the prognostic uncertainty they had experienced since the prenatal diagnosis and the subsequent progression and fluctuation of respiratory symptoms. We explained that repeated cyanotic spells are life-threatening and cause suffering for the patient, so they should be avoided regardless of the place of care. We also explained that home care is much more suitable for the child's quality of life and developmental outcome. After several conversations, the parents decided to have their child undergo a tracheostomy. The procedure was carried out on DOL 140.

**Figure 4:**
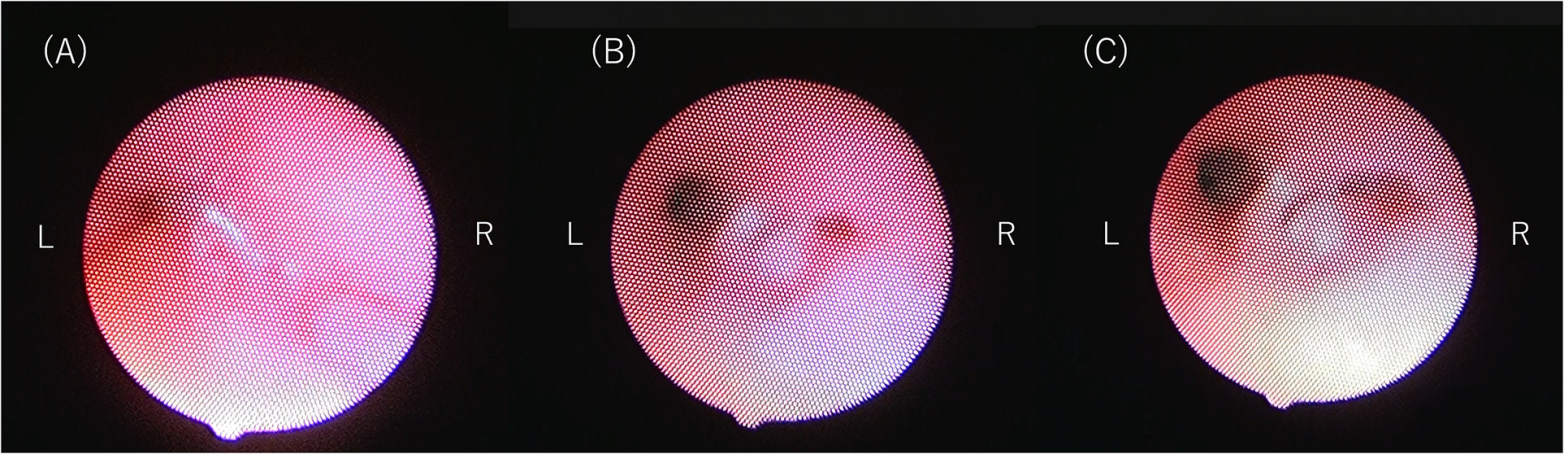
**(A)** bronchoscopy image showing complete tracheal collapse. **(B)** Bronchoscopy image at inspiratory pressure of 14 cm H₂O and a PEEP of 7 cm H₂O. **(C)** Bronchoscopy image at inspiratory pressure of 20 cm H₂O and a PEEP of 7 cm H₂O. Note the improved patency of the left bronchus compared to **(C)**. The right bronchus remains narrowed due to congenital hypoplasia of the right lung and bronchus. There is no evidence of tracheal rings or external compression of the trachea.

Bronchoscopy on DOL 191 (Figure 4) showed that the trachea and left bronchus had completely collapsed upon straining, which is consistent with a severe TBM diagnosis. High PEEP alone could not prevent the collapse, and the patient required a set inspiratory pressure of more than 20 cm H₂O (relative to atmospheric pressure) to maintain lumen patency throughout the respiratory cycle. Due to congenital hypoplasia of the right lung and bronchus, a fixed stenosis of the right bronchus was also identified, consistent with the CT image. There was no evidence of tracheal rings.

Although higher inspiratory pressures maintain better lumen patency, we initially set the therapeutic mechanical ventilation settings to an inspiratory pressure of 20 cm H₂O and a PEEP of 7 cm H₂O. This setting was chosen based on the bronchoscopic findings to prevent complete airway collapse while minimizing the risk of left lung hyperinflation. Despite his delicate anatomical condition, the patient's respiratory condition remained stable with these settings. In response to the parents’ expressed preference for home-based care, a coordinated discharge plan was developed through discussions with the medical team. After several in-hospital overnight stays, the patient was discharged on DOL 219.

At nine months of age, the patient's physical and mental development was normal. He continues to be managed with home mechanical ventilation, but gradually spends more time with a speech valve. Figure 1D shows a chest x-ray after discharge. He has not developed respiratory failure requiring hospitalization.

## 3 Discussion

The mortality rate of CDH with HPF is as high as 40%–50% (1). Challenges in the management of CDH with HPF include difficulty in preoperative diagnosis, difficulty in achieving a safe surgical plane, and a high incidence of cardiovascular comorbidities. In fatal cases, most deaths occur either during or within 2 weeks of surgery (1, 2), making the prevention of surgical comorbidities or the choice of approach or timing according to the patient's tolerance of surgical invasion of paramount importance. While complete vs. partial or even no separation of HPF has always been controversial in terms of achieving a better prognosis, a few recent reports have focused more on the importance of planning individualized approaches and timing for surgery after careful evaluation of patients’ anatomical characteristics, leading to a two-stage surgical approach (3–5).

The importance of evaluating and managing pulmonary hypertension and abnormal vascular communication is well-documented, but airway involvement has never been reported, as shown in Table 1. The present case highlights the importance of assessing airway involvement. Because many children with primary TBM do not show symptoms until the age of 2–3 months, distinguishing whether the patient's TBM is primary or acquired is difficult. However, we speculate that prolonged intubation and excessive breathing work after extubation due to right lung hypoplasia were complicit in the development of severe TBM. Although TBM has not been reported in patients with HPF to date, our case is noteworthy considering that the intubation period is more extended with a two-stage than with a single-stage surgical approach.

**Table 1:** Survival cases managed with two-stage surgical approaches for hepatopulmonary fusion.

Authors	Operation date	Clinical course	Outcome
Tanaka et al. (2006) (3)	4 days of age 13 months of age	During the first operation, only a plication of the hernia sac was performed because the liver and the lung both tightly adhered to the posterior part of the diaphragm. Without a prominent improvement of the respiratory condition, a tracheostomy was placed on DOL 129. A cardiopulmonary angiography at 1 year of age showed prominent pulmonary hypertension, the right pulmonary venous draining into the liver, and an extended feeder vessel from the hepatic artery to the right lung. During the second operation, the aberrant vessels between the right lung and the liver were carefully ligated, and a hepatic segmentectomy of S6 and S7 was performed.	Doing well for 21 months without any mechanical ventilation since 2 months after the radical operation.
Franco et al. (2023) (4)	2 days of age 4 months of age	The patient was diagnosed with type D CDH and HPF during the first operation. The liver also adhered to the mediastinum in the vicinity of the great vessels. Partial reduction of the liver into the abdominal cavity and plication of the hernia sac were performed. A postoperative echocardiogram showed no vascular abnormalities except for a hypoplastic right pulmonary artery. After managing severe pulmonary hypertension and patent ductus arteriosus, the patient underwent a second surgical session at 4 months of age. Leaving the liver and the mediastinum adhered, complete tissue division of the lung and the liver was performed. The patient was discharged at six months of age.	At the three-year-old follow-up, the patient does not require any oxygen therapy for daily life.
Cain-Trivette et al. (2024) (5)	3 days of age 14 days of age	During the first operation, the bowel was reduced to the abdomen, but the liver could not be reduced because of HPF. Postoperatively, the patient had worsening pulmonary hypertension. A CTA confirmed the presence of HPF with a right-sided intra-lobar sequestration and a single right pulmonary vein. During the second operation, the separation of the HPF was forgone due to the right pulmonary vein coursing immediately adjacent to the HPF. The diaphragmatic defect was closed by sewing the diaphragm around the liver. The patient was extubated on DOL 31 and was discharged on DOL 138.	Not mentioned.
Kanda et al. (2025)	2 days of age 28 days of age	The patient was diagnosed with type C CDH and HPF during the first operation. HPF was left unseparated, and only the bowel was reduced to the abdominal cavity. A CT scan showed no abnormal vascular communication. In the second operation, HPF was carefully separated with minimal bronchopulmonary resection. The patient was extubated on DOL 41 but suffered from severe tracheobronchomalacia. Tracheostomy was placed on DOL 133 and the patient was discharged on DOL 219 with home mechanical ventilation.	At 9 months old, physical and mental development is appropriate for his age. Managed with home mechanical ventilation, the patient has not suffered from respiratory failure requiring hospital admission.

Our case is unique in terms of TBM development in a patient with an almost unilateral lung, where the liver occupied the right hemithorax. External compression should always be considered when evaluating the cause of a TBM. In our case, tracheal compression by the liver was not remarkable on postoperative CT or bronchoscopy. However, due to the elevated liver in the right thorax, aortopexy or external stenting was not indicated. Since TBM is rarely reported in patients with unilateral lung aplasia or hypoplasia, no consensus regarding the management and prognosis of such patients has been established to date. Nevertheless, our case suggests that bronchoscopy remains the gold standard for evaluating TBM severity and planning therapeutic strategies, as it visualizes the dynamic features of the airway. Although the patient remains on home mechanical ventilation, our case is remarkable in that the patient's physical and mental development was age-appropriate, without additional invasive surgery or respiratory deterioration over a period of months.

One of the challenges in this case is communicating with the parents of a patient with a rare congenital condition and an uncertain prognosis. It is reported that neonatologists tend to be pessimistic, while parents prefer an optimistic framework and have an optimistic bias (7, 8). Parents of patients admitted to the NICU often experience uncertainty, and intolerance of uncertainty has been linked to parental psychological well-being (9). Parents of patients with rare diseases report not only emotional and psychological needs but also social, informational, practical, and physical needs (10). Physicians should be aware of these factors, reflect on their communication with parents, and provide holistic support in cooperation with other professionals.

In conclusion, our case emphasizes the importance of evaluating the patient's preoperative anatomical and physiological characteristics when managing CDH with HPF. To achieve this goal, a two-stage surgical approach may be safer than a single-stage approach. Nevertheless, TM/TBM should be considered after management involving long-term intubation in these cases.

## Data Availability

The original contributions presented in the study are included in the article/Supplementary Material, further inquiries can be directed to the corresponding author.

## References

[1] Ferguson DM, Congenital Diaphragmatic Hernia Study Group. Hepatopulmonary fusion: a rare variant of congenital diaphragmatic hernia. J Pediatr Surg. (2020) 55(9):1903–7. 10.1016/j.jpedsurg.2019.09.037 31708208

[2] Rocha GMD. Congenital hepatopulmonary fusion. Eur J Pediatr Surg. (2022) 32(6):477–96. 10.1055/s-0042-1749213 36027900

[3] Tanaka S Kubota M Yagi M Okuyama N Ohtaki M Yamazaki S Treatment of a case with right-sided diaphragmatic hernia associated with an abnormal vessel communication between a herniated liver and the right lung. J Pediatr Surg. (2006) 41(3):e25–8. 10.1016/j.jpedsurg.2005.12.032 16516610

[4] Franco MA Alzate-Ricaurte S Alzate Gallego ED Kafury DF Botero ALG Avila DC. Survival after a two-stage surgical approach in hepatopulmonary fusion: a case report. Int J Surg Case Rep. (2023) 108:108467. 10.1016/j.ijscr.2023.108467 37423148 PMC10382799

[5] Cain-Trivette CJ Robinson NB Kadenhe-Chiweshe A Steigman S Spigland NA. A novel approach for repair of right sided congenital diaphragmatic hernia and hepatopulmonary fusion. J Surg Case Rep. (2024) 2024(9):rjae566. 10.1093/jscr/rjae566 39239144 PMC11374373

[6] Morini F Lally PA Lally KP Bagolan P. The congenital diaphragmatic hernia study group registry. Eur J Pediatr Surg. (2015) 25(6):488–96. 10.1055/s-0035-1569151 26642385

[7] Nayak B Moon JY Kim M Fischhoff B Haward MF. Optimism bias in understanding neonatal prognoses. J Perinatol. (2021) 41(3):445–52. 10.1038/s41372-020-00773-1 32778685

[8] Callahan KP Munson D Feudtner C. A cure for prognostic pessimism among neonatologists. JAMA Netw Open. (2024) 7(2):e240525. 10.1001/jamanetworkopen.2024.0525 38393732

[9] Rambod M Pasyar N Mazarei Z Soltanian M. The predictive roles of parental stress and intolerance of uncertainty on psychological well-being of parents with a newborn in neonatal intensive care unit: a hierarchical linear regression analysis. BMC Pediatr. (2023) 23(1):607. 10.1186/s12887-023-04420-4 38037025 PMC10691133

[10] Pelentsov LJ Laws TA Esterman AJ. The supportive care needs of parents caring for a child with a rare disease: a scoping review. Disabil Health J. (2015) 8(4):475–91. 10.1016/j.dhjo.2015.03.009 25959710

